# The efficacy of a phone assistance nursing program for functional outcomes in patients after shoulder instability surgery: A protocol for randomized controlled trial

**DOI:** 10.1097/MD.0000000000022756

**Published:** 2020-10-23

**Authors:** Yongling Zheng, Hongli Wang, Huali Wang, Junchang Xu, Ping Chen

**Affiliations:** a Department of Orthopedics; b Department of Operating Room; c Department of Gynaecology and Obstetrics, the First People's Hospital of Xiangyang City Affiliated to Hubei Medical College, Hubei, China.

**Keywords:** function, phone assistance nursing program, protocol, shoulder instability surgery

## Abstract

**Objective:**

We conduct this research protocol for the assessment of the effect of phone-assisted care programs on functional outcomes in patients receiving shoulder instability surgery.

**Methods:**

This is a randomized controlled, single center trial which will be implemented from October 2020 to December 2021. This trial is conducted according to the SPIRIT Checklist of randomized researches. It was authorized via the Ethics Committee of the First People's Hospital of Xiangyang city affiliated to Hubei Medical College (XY234-026). Ninety participants who undergo shoulder instability surgery are analyzed. Patients are randomly divided into control group (standard management group, with 45 patients) and study group (the phone program group, with 45 patients). In control group, the exercises at home are not monitored. Whereas in study group, patients are asked about their at-home activities, and the extra coaching sessions are provided to patients on self-care, exercise guidance, and the importance of exercise at home, and then answers to their questions. The primary outcome is the range of motion of the shoulder joint, and the pain arcs are determined through the range of motion. The extra assessments include the shoulder functional outcome, pain, and the quality of life. All the analysis needed in this study is implemented with SPSS (IBM, Chicago, USA) for Windows Version 19.0.

**Results:**

The clinical outcome variables between groups are shown in Table.

**Conclusion:**

This investigation can offer a reliable basis for the effectiveness of phone assistance nursing program in patients after shoulder instability surgery.

**Trial registration number:**

researchregistry6010

## 1 Introduction

Shoulder instability is one of the most familiar clinical manifestations of sports trauma.^[1,2]^ The structure of glenohumeral joint is unstable, which is the most common dislocation joint.^[3]^ The dislocation of anterior shoulder is the most familiar type, which accounts for 90%.^[4]^ In general population, the incidence of shoulder dislocation is between 12.3 and 26.2 per 100,000 person-years.^[5]^ Patients range in age, but are most familiar among young people while rare in children. At present, the most common treatment for the simple first shoulder dislocation is non-surgical treatment.^[6]^ Nevertheless, several articles reported a high incidence of poor functional outcome.^[7,8]^ More and more studies have recommended that surgical procedure is an alternative choice for shoulder instability.^[9,10]^ The rehabilitation after operation is a necessary condition for the recovery of shoulder joint activity. After the sling removed, the management after operation usually involves supervised physical therapy in the outpatient department, and supplemented by the unsupervised exercises at home.

Therefore, the purpose of surgery and follow-up treatment is to improve the patients’ functional outcomes and the quality of life. Nevertheless, about 25% of the patients had limited shoulder joint movement, mainly external rotation, and less physical activity after operation.^[11]^ One reason for the lack of success may be pain and the patients’ fear of conducting shoulder exercises immediately after the sling is removed, which can result in a lack of adherence to home exercises and longer periods of physical therapy at clinic. Home-based physiotherapy, telemedicine support after discharge, and phone counseling, have been used for these orthopedic problems.^[12,13]^ However, few study have reported the use of phone assistance nursing program for patients after shoulder instability surgery. Therefore, we conduct this research protocol for the assessment of the effect of phone-assisted care programs on the functional outcomes in patients receiving shoulder instability surgery.

## 2 Methods

### 2.1 Study design

This is a single center and randomized controlled trial which will be implemented from October 2020 to December 2021. This trial is conducted according to the SPIRIT Checklist of randomized researches. It was authorized via the Ethics Committee of the First People's Hospital of Xiangyang city affiliated to Hubei Medical College (XY234-026), and it has been registered in the research registry (researchregistry6010).

### 2.2 Patients and randomization

Ninety participants who undergo shoulder instability surgery are analyzed. In the random envelope, all patients are assigned a random number via using the random number table, and the result of allocation is hidden. Patients are randomly divided into control group (standard management group, with 45 patients) and study group (the phone program group, with 45 patients). The inclusion criteria is as follows:

(1) the repair of arthroscopic Bankart and the recurrent anterior instability;
(2) the age of people varies from 40 years to 60 years.

Exclusion criteria is composed of:

(1) previous shoulder involvement, multidirectional or posterior instability, and the prior rotator cuff injury symptoms;
(2) patients with the history of severe renal and hepatic dysfunction. The magnetic resonance imaging and radiography are applied to evaluate the related lesions.

### 2.3 Interventions

All the operations are carried out through a same surgeon in lateral position under the condition of intervertebral nerve block and general anesthesia. All the patients undergo the repair of standard arthroscopic Bankart with the suture anchors. After the sling is removed, the 2 groups are given physiotherapy for approximately 1 month in our hospital outpatient department. When the range of motion (ROM) of the shoulder joint reaches a completely painless state or cannot be further improved, the physiotherapy is stopped. In accordance with the standard practice, all the patients in the 2 groups are given written and verbal information asking them to conduct the daily exercises and activities at home as the supplement to outpatient rehabilitation. The written information involves a handout with advice on upper body exercises and instructions for the home exercises, for instance, carrying weights on hands and limiting extreme exercise. In control group, the exercises at home are not monitored. Whereas in study group, patients are asked about their at-home activities, and the extra coaching sessions are provided to patients on self-care, exercise guidance, and the importance of exercise at home, and then answers to their questions.

### 2.4 Evaluations

The clinical assessment is implemented for the patients before and after the operation at 1 month, 2 months, 6 months, and 12 months. The primary outcome is the ROM of the shoulder joint, and the pain arcs are determined through the ROM. The extra assessments include the shoulder functional outcome, pain, and the quality of life. For the measurement of pain, the visual analogue scale^[14]^ is utilized (0 represents no pain; and 10 represents most severe possible pain). The Oxford shoulder instability score^[15]^ is applied for the evaluation of the shoulder function result. Oxford shoulder instability score is an effective questionnaire to evaluate the prognosis of patients with shoulder instability. It is composed of twelve items that record daily life activities (including the emotional and physical states, leisure, sports as well as lifestyle). The score ranges from 12 (with no difficulty and excellent results) to 60 (with maximum difficulty and poor results). For the measurements of life quality, it is conducted with disability of the arm, shoulder and hand (DASH) (the hand, shoulder, and arm disability score).^[16]^ DASH is a kind of quality of life questionnaire related to health, which is extensively utilized in the patients with any upper extremity disease internationally. DASH possesses 11 questions that address functions, symptoms, and psychological as well as social aspects. And it's scores are between 11 (represents no disability) and 55 (represents the highest disability).

### 2.5 Statistical analysis

All the required analyses are carried out via applying the SPSS (IBM, Chicago, USA) for Windows Version 19.0. All data are expressed with appropriate characteristics, for instance, mean, median, standard deviation as well as percentage. The comparison between the 2 groups are conducted through applying the independent samples *t* test or the Mann–Whitney *U* test. And for the comparison of categorical variables between the groups, it can be implemented with Chi-squared test. When the *P* value is less than .05, it is regarded as a significant in statistics.

## 3 Results

The clinical outcome variables between groups are shown in Table 1.

**Table 1:**
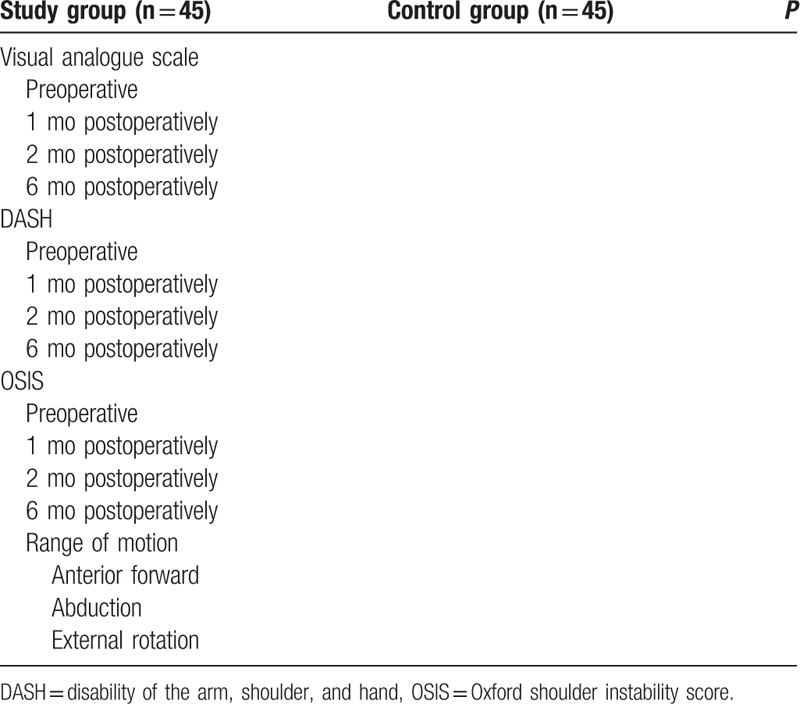
Outcome measures between study group and control group.

## 4 Discussion

To the best of our knowledge, this is the first randomized controlled study for the analysis of the results of physiotherapy based on family in the patients undergoing shoulder instability surgery. Shoulder instability is an abnormal condition in which the humeral head slips out of glenoid cavity.^[17]^ The anterior instability of shoulder joint is the result of primary dislocation, accompanied by the related diseases, for instance, the Bankart disease.^[18]^ In the shoulder diseases, clinical results may be affected through the biopsychosocial features, patient expectations or the personality traits. No program supported by postdischarge phone is utilized for the shoulder instability. Successful outcomes for body function and knee function are reported after a phone consultation program after discharge in patients undergoing total knee arthroplasty. Another research that guided patients with the radial head fractures found that this kind of intervention enhanced functional outcomes and ROM from preoperative to ultimate follow-up. In our investigation, the sample size we used is relatively small. In order to confirm the results, the larger investigations is required.

## 5 Conclusion

This investigation can offer a reliable basis for the effectiveness of phone assistance nursing program in patients after shoulder instability surgery.

## Author contributions

Ping Chen plan the study design. Junchang Xu review the protocol. Hongli Wang and Huali Wang collect data. Yongling Zheng write the manuscript. All authors approve the submission.

**Data curation:** Junchang Xu.

**Formal analysis:** Hongli Wang.

**Funding acquisition:** Ping Chen.

**Methodology:** Huali Wang.

**Software:** Junchang Xu.

**Writing – original draft:** Yongling Zheng.
